# Tryptophan-sorbitol based carbon quantum dots for theranostics against hepatocellular carcinoma

**DOI:** 10.1186/s12951-022-01275-2

**Published:** 2022-02-14

**Authors:** Yang Wang, Jun Chen, Jiekang Tian, Guanchen Wang, Weikang Luo, Zebing Huang, Yan Huang, Ning Li, Mingming Guo, Xuegong Fan

**Affiliations:** 1 Key Laboratory of Viral Hepatitis of Hunan Province, Xiangya Hospital, Central South University, Xiangya Road 87, Kaifu District, Changsha, 410008 China; 2 Institute of Integrative Medicine, Department of Integrated Traditional Chinese and Western Medicine, Xiangya Hospital, Central South University, Changsha, 410008 China; 3 National Clinical Research Center for Geriatric Disorders, Xiangya Hospital, Central South University, Changsha, 410008 China; 4 Department of Infectious Diseases, Xiangya Hospital, Central South University, Changsha, 410008 China; 5 School of Chemistry and Chemical Engineering, Southwest University, Tiansheng Road, Beibei District, Chongqing, 400715 China; 6 Department of Blood Transfusion, Xiangya Hospital, Central South University, Changsha, 410008 China

**Keywords:** Tryptophan, Sorbitol, Carbon quantum dots, Theranostics, Hepatocellular carcinoma

## Abstract

**Background:**

Despite novel advances in screening, targeting and immunotherapies, early diagnosis and satisfactory treatments against hepatocellular carcinoma (HCC) remain formidable challenges. Given the unique advantages, carbon quantum dots (CQDs) become a smart theranostic nanomaterial for cancer diagnosis and therapy.

**Results:**

In this work, a type of bio-friendly CQDs, trichrome-tryptophan-sorbitol CQDs (TC-WS-CQDs), is synthesized from natural biocompatible tryptophan via the one-pot hydrothermal method. Compared with normal hepatocytes, a much stronger green fluorescence is detected in HCC cells, indicating the ability of TC-WS-CQDs to target HCC cells. Furthermore, green-emitting TC-WS-CQDs generate large amounts of reactive oxygen species (ROS), leading to autophagy of HCC cells. Additionally, the green-emitting TC-WS-CQDs perform significant tumor inhibition by inducing autophagy via p53-AMPK pathway in vitro and in vivo studies with almost no systemic toxicity.

**Conclusions:**

The results may highlight a promising anticancer nanotheranostic strategy with integration of diagnosis, targeting, and therapy.

**Graphical Abstract:**

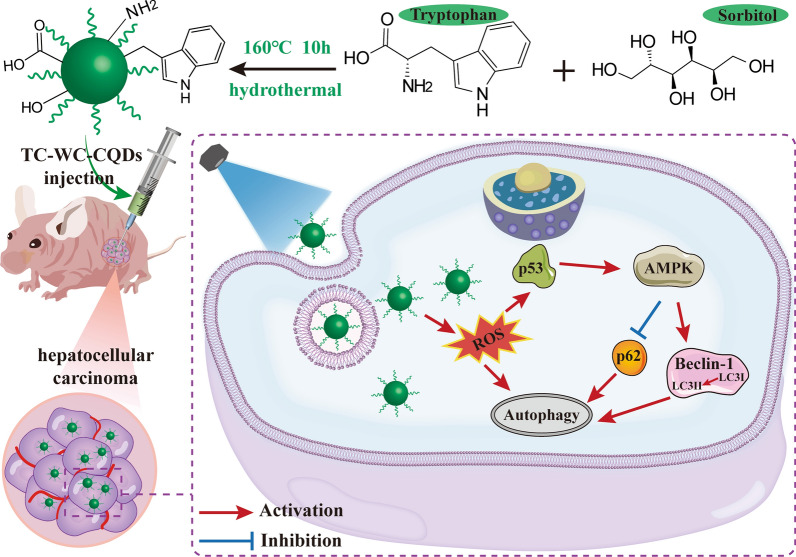

**Supplementary Information:**

The online version contains supplementary material available at 10.1186/s12951-022-01275-2.

## Introduction

Liver cancer is the sixth ranked cancer, as well as the third leading cause of cancer death worldwide in 2020, with approximately 906,000 new cases and 830,000 deaths [1]. Among all types of liver cancer, hepatocellular carcinoma (HCC) accounts for 75–85% of the total cases [1]. Despite novel advances in screening, targeting and immunotherapies, early diagnosis and satisfactory treatments remain formidable challenges [2]. The overall mortality is up to 95% because of the low percentage of radical resectable HCC on diagnosis [3]. Although liver transplantation tends to be an optimal treatment, critical shortage of available liver allografts limits the application [4]. Even the new-generation drugs merely make the median overall survival continue to be 1 year [5]. Current randomized trials of immune checkpoint inhibitor therapy, such as anti-programmed cell death protein 1 (anti-PD-1) and anti-programmed cell death-ligand protein 1 (anti-PD-L1) monotherapy, do not demonstrate significant improvement in overall survival against HCC [6]. More potential screening tests and efficacious therapies to prolong mean survival are extremely urgent [4].

Considering limitations of standard diagnosis and therapeutics, we realize that the dominant “one-size-fits-all” diagnostic and therapeutic agents for cancer treatment have heralded the need for “personalized medicine” [7]. Personalized medicine aims to maximize therapeutic efficacy with minimal delay after diagnosis [8]. To achieve this goal, theranostics is focused, which offers promising prospects in personalized medicine. Theranostics is a single platform to combine diagnosis and therapy, not only provides precise information on position, type and size of tumors, but also functions as preferable therapeutics [9]. Thus, theranostics has emerged as a hot spot research in cancer treatment [10].

With frontier developments of nanotechnology in medical field [11–13], scientists are striving to explore nanotheranostic strategy for HCC prevention [14]. To improve theranostic efficiency, lots of drug delivery nanosystems have been developed [15–17]. Of these functionalized nanomaterials, carbon quantum dots (CQDs) attract great research attention due to their unique advantages, including ultrasmall size, high water-solubility, excellent photoluminescence (PL), broad emission range and outstanding biocompatibility [18, 19]. Additionally, CQDs, as a class of zero-dimensional carbon nanomaterials, contain many functional groups such as epoxy, carbonyl, hydroxyl, amine, and carboxyl on their surfaces and/or edges, which give rise to high hydrophilicity and readiness for functionalization with biological species [20, 21]. Given these properties, CQDs become a smart theranostic nanomaterial to exhibit tumor identification and enhance anticancer effects.

There have been numerous studies on exploration of CQDs for cancer diagnosis and therapy [22–24]. However, most of these studies focused on either fluorescent diagnosis or anticancer activity, few concentrated on the intrinsic theranostic applications [19]. Moreover, CQDs may irradiate with light degrade into molecules that are toxic to both normal and malignant cells [25]. Therefore, it is imperative to design synthetic CQDs for theranostics using natural carbon sources as precursors without organic solvents.

**Scheme 1:**
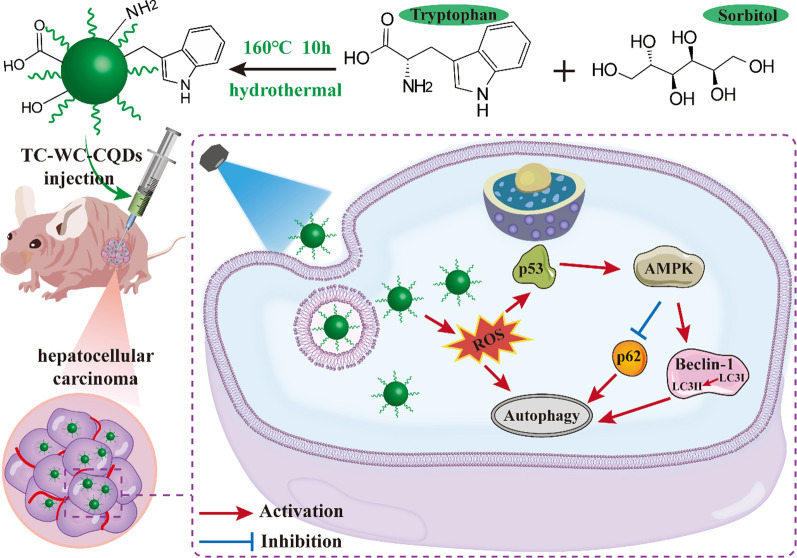
Schematic illustration of the synthesis and light-induced antitumor mechanism of the TC-WS-CQDs

Herein, we fabricate hybrid fluorescent trichrome-tryptophan-sorbitol CQDs (TC-WS-CQDs) from natural biocompatible tryptophan via a green hydrothermal method. The formed CQDs (~ 1.4 nm) include conjugated structure induced by tryptophan and scaffold structure formed by sorbitol, which exhibit three-color emission (blue, green, and red). Compared with normal hepatocytes, we found that TC-WS-CQDs were more willing to be selectively delivered into HCC cells by endocytosis. The TC-WS-CQDs exhibited strong visible blue, green and red fluorescence in tumor cells under different excitation wavelengths. Furthermore, we found that green-emitting TC-WS-CQDs generated large amounts of reactive oxygen species (ROS), leading to autophagy of HCC cells. In addition, these green-emitting TC-WS-CQDs performed significant tumor inhibition by inducing autophagy via p53-AMPK pathway in vitro and in vivo studies with almost no systemic toxicity. The results may highlight a promising anticancer nanotheranostic strategy with integration of diagnosis, targeting, and therapy (Scheme 1).

## Methods

### Materials

Tryptophan and 2,2,6,6-tetramethylpiperidine were purchased from Sigma-Aldrich (St.Louis, MO, USA). Sorbitol was supplied by Aladdin Biochemical Technology Co., Ltd (Shanghai, China). Dulbecco’s Modified Eagle’s Medium (DMEM, high glucose), Roswell Park Memorial Institute (RPMI) 1640 medium, and 10% fetal bovine serum (FBS) were purchased from Naer Biotechnology Co., Ltd (Tianjin, China). LysoTracker Red was obtained from Maokang Biotechnology Co., Ltd (Shanghai, China). Cell Counting Kit-8 (CCK8) was obtained from Vazyme Biotech Co., Ltd (Nanjing, China). The 3-(4,5-dimethylthiazol-2-yl)-5-(3-carboxymethoxyphenyl)-2-(4-sulfophenyl)-2H-tetrazolium, inner salt (MTS) was obtained from Promega Corporation (USA). Reactive Oxygen Species Assay Kit was supplied by Beyotime (Shanghai, China). Nystatin, Chlorpromazine, *N*-acetylcysteine (NAC) and Apocynin (APO) were obtained from Selleck. cn (Shanghai, China). mCherry-GFP-LC3 fusion protein were purchased from Tsingke Biotechnology Co., Ltd (Beijing, China).

### Synthesis of TC-WS-CQDs

Tryptophan (100 mg, 0.49 mmol) and sorbitol (500 mg, 2.74 mmol) were dissolved in water and diluted to 50 ml, and the mixture was divided into ampoules of 5 ml capacity, which was sealed and heated to 160 °C for 10 h. After cooling to room temperature, the ampoules were stored at 4 °C for future use.

### Synthesis of blue (B), green (G) and red (R)-WS-CQDs

TC-WS-CQDs (100 ml) were centrifuged at 8000*g* for 10 min and the supernatant was lyophilized. The lyophilized powder was purified by silica-gel column chromatography gradient elution, starting from ethyl acetate: ethanol = 10:1, and gradually increasing the polarity of the eluent to ethyl acetate: ethanol = 1:1. Depending on the separation effect, the silica-gel column chromatography process may need to be repeated two or more times to obtain the blue (B), green (G) and red (R)-WS-CQDs.

### Cellular imaging and transmission electron microscopy

In the cell image experiments, the hepatoma cells (Huh7 cells) were seeded into 24-well plates with cell climbing slice and cultured overnight. Then, cells were incubated with TC-WS-CQDs (100 μg/ml) for 6 h. Next, cells were washed with phosphate buffered saline (PBS) three times and fixed with 4% paraformaldehyde for 15 min. After that, images were acquired with a laser scanning confocal microscopy (LSCM) under the excitation wavelengths of 405 nm, 488 nm, and 545 nm. For the transmission electron microscopy (TEM) analysis, after incubation with TC-WS-CQDs for 6 h, trypsinized Huh7 cells were fixed with 3% glutaraldehyde in phosphate-buffered saline and then were sent to the Department of Pathology in Xiangya Hospital for further processing and scanning.

### Lyso-Tracker Red staining

Lysosomal staining was performed using Lyso-Tracker Red (Invitrogen, L7528). Huh7 cells were seeded into 24-well plate with cell climbing slice. After 6 h of treatment with TC-WS-CQDs (100 μg/ml), cells were incubated with LysoTracker Red at 75 nM for 30 min at 37 °C and washed three times with PBS. Then, cells were fixed with 4% paraformaldehyde and photographed with fluorescence microscope under the excitation wavelength of 545 nm.

### Cytotoxicity assay

Cell viability was estimated by CCK8 and MTS assay. Huh7 cells and the normal liver cells (L02 cells) were seeded into 96-well plates at a density of 8 × 10^3^ cells/well overnight, followed by incubation with TC-WS-CQDs with different concentrations (0, 50, 100, 200 μg/ml). The media with non-cells was blank control. After incubation with different time (12 h, 24 h, 48 h), 10 μl CCK8 (Vazyme, A311-02) or 20 μl MTS (Promega, G3582) solution were added to each well for 2 h. Finally, the optical density (OD) of the wells was measured at 450 nm (CCK8) and 490 nm (MTS) by a microplate reader. Based on the OD value, cell viability was calculated: cell viability (%) = (OD_treated_ − OD_blank_)/(OD_control_ − OD_blank_) × 100% (OD_control_, OD_treated_ and OD_blank_ were the values obtained without or with TC-WS-CQDs and blank control, respectively).

### Antitumor effects of TC-WS-CQDs in vitro

#### Proliferation assay

In the cell proliferation experiments, Huh7 cell suspensions with TC-WS-CQDs (75 μg/ml) or without TC-WS-CQDs were continuously exposed to or not exposed to light (470 nm or 545 nm) equipped with fluorescence microscope for 10 min. After different treatment, 100 μl cell suspensions were transferred to 96-well plates (5 × 10^3^ cells/well), and 10 μl CCK8 or 20 μl MTS solution was added to each well after culturing for 0 h, 24 h, 48 h, 72 h and 96 h. Following incubation for another 2 h, the OD value was assessed as previously described in cytotoxicity assay.

#### Wound healing assay

Cell migration capacity was measured by wound healing assay. ~ 4 × 10^5^ Huh7 cells with different treatment were seeded into 6-well plates and cultured in DMEM with 10% FBS. After cells reached 90–100% confluence, a line wound was scratched by a 10 μl tip and the detached cells were removed by washing with PBS. Subsequently, the cells were cultured in serum-free DMEM. The wounds were observed at 0 h, 24 h, 48 h and 72 h. The wound healing rate was calculated according to the following formula: Wound healing rate = [(wound width at 0 h) − (wound width at each time point)]/(wound width at 0 h) × 100%.

#### Reactive oxygen species and singlet oxygen measurement

Intracellular production of ROS was determined using Reactive Oxygen Species Assay Kit (Beyotime, S0033). After 24 h incubation with different treatment, cells were incubated with 10 μM 2′,7′-dichlorodihydrofluorescein diacetate dye (DCFH-DA) in DMEM for 30 min. Then, cells were washed with PBS, trypsinized and resuspended in PBS. The green fluorescence (DCFH-DA), corresponding to ROS levels, was determined using flow cytometer, microplate reader and fluorescence microscope. *N*-acetylcysteine (Selleck, S1623) and Apocynin (Selleck, S2425) were used as ROS inhibitors in this research.

For the singlet oxygen (^1^O_2_) detection, three samples of TC-WS-CQDs aqueous solution containing 1% 2,2,6,6-tetramethylpiperidine (TEMP) and one sample of 1% TEMP aqueous solution were treated by avoiding light for 30 min, 470 nm laser irradiation for 5 min, and 30 min respectively, and then transferred to quartz capillary for electron spin resonance (ESR) spectra measurement.

#### Western blot analysis

Cells were collected and lysed in strong RIPA buffer at 4 ℃ for 1 h. Samples were subsequently centrifuged at 12,000 rpm for 15 min at 4 ℃ and the supernatants were collected. The protein was quantified using Pierce BCA ProteinAssay (Thermo Scientific, USA, 23228). Protein was diluted in SDS-PAGE loading buffer (YEASEN, S8901110) and denatured at 95 ℃ for 10 min. Equal amounts of samples were separated by 10–12% SDS-PAGE and transferred onto PVDF membranes. After blocking with 5% non-fat milk for 1 h at room temperature, the membranes were incubated with the indicated antibodies (LC3B, 1:2000, SigmaL7543; Beclin1, 1:1000, CST4122; p62, 1:1000, CST88588; p53, 1:500, CST2524; AMPK, 1:1000, CST5831; pAMPK, 1:1000, CST2535; BAX, 1:1000, CST2772; Bcl-xL, 1:1000, CST2764; Caspase-3, 1:1000, CST14220; GAPDH, 1:1000, SC-47724; β-actin, 1:1000, SC-69879) overnight at 4 ℃. Membranes were washed with TBST three times and incubated in secondary antibodies (HRP-conjugated goat anti-mouse antibody, 1:3000, ab6789; HRP-conjugated goat anti-rabbit antibody, 1:3000, ab6721) for 1.5 h at room temperature. The blots were finally detected using an enhanced chemiluminescence system.

#### mCherry-GFP-LC3 transient transfection

For autophagic flux analysis, Huh7 cells with different treatment were infected with adenovirus expressing mCherry-GFP-LC3 fusion protein. After incubation in complete medium for 24 h, cells were observed under a fluorescence microscope. Autophagic flux was assessed by manually counting the number of yellow and red dots of each cell in five random fields from the images that merged the red and green channels. Yellow and red dots represented autophagosomes and autolysosomes, respectively.

### Vivo experiment

#### Evaluation of vivo toxicity

For the in vivo toxicity, BALB/nu male mice (6 weeks old) were purchased from Changzhou Cavens Laboratory Animal Company or C57BL/6J male mice (6 weeks old) were purchased from Hunan SJA Laboratory Animal Company. After 1 week acclimation, the mice were randomly divided into two groups and were treated with 300 μl (750 μg) TC-WS-CQDs or 300 μl PBS by tail vein injection every 2 days. Blood samples were collected for the toxicity-related parameters analysis and major organs were fixed with 4% paraformaldehyde for hematoxylin–eosin (HE) staining at day 14.

#### Photodynamic therapy (PDT) in hepatocellular carcinoma bearing mice

BALB/nu mice (5 weeks) were purchased from Changzhou Cavens Laboratory Animal Company. Following 1 week acclimation, hepatocellular carcinoma was established by subcutaneous injection of 2.5 × 10^6^ of Huh7 cells suspended in 100 μl of PBS into the nude mice. The mice were randomly divided into four groups with each 5 mice: CQDs + I_470nm_ group (injection of TC-WS-CQDs through tail vein and 470 nm irradiation for 10 min), CQDs group (injection of TC-WS-CQDs through tail vein), I_470nm_ group (470 nm irradiation for 10 min), PBS group (injection of PBS through tail vein). The mice were subjected to different treatment every 2 days when the tumor size reached 5–8 mm. The body weight and the tumor size were monitored every 2 days.

#### Immunohistochemistry

The mice were sacrificed and tumors were extracted at day 10. Immunohistochemical detection was performed using the DAKO kit (K5007, Denmark) following the manufacturer’s instructions. The primary antibody LC3 was 1:300 dilution (14600-1-AP, Proteintech), primary antibody p53 was 1:200 dilution (21891-1-AP, Proteintech), and primary antibody pAMPK was 1:100 dilution (CY6027, Abways). The secondary antibody was Goat-anti-Rabbit IgG (DAKO). After immunostaining, sections were counterstained with hematoxylin.

### Statistical analysis

Statistical graphs were plotted by OriginPro 2021 software or GraphPad Prism 8.0.1 software. Statistical analysis was performed by SPSS 22.0 software. Data were represented as means ± SD. Comparisons of data with a normal distribution between two groups were analyzed using unpaired t tests or two-way ANOVA. Otherwise, comparisons were analyzed using nonparametric Mann–Whitney test. p value less than 0.05 was considered statistically significant. ****p < 0.0001, ***p < 0.001, **p < 0.01, *p < 0.05, ns: not significant. All experiments were performed at least in triplicate.

## Results and discussion

### Synthesis and structural characterizations of TC-WS-CQDs

**Fig. 1:**
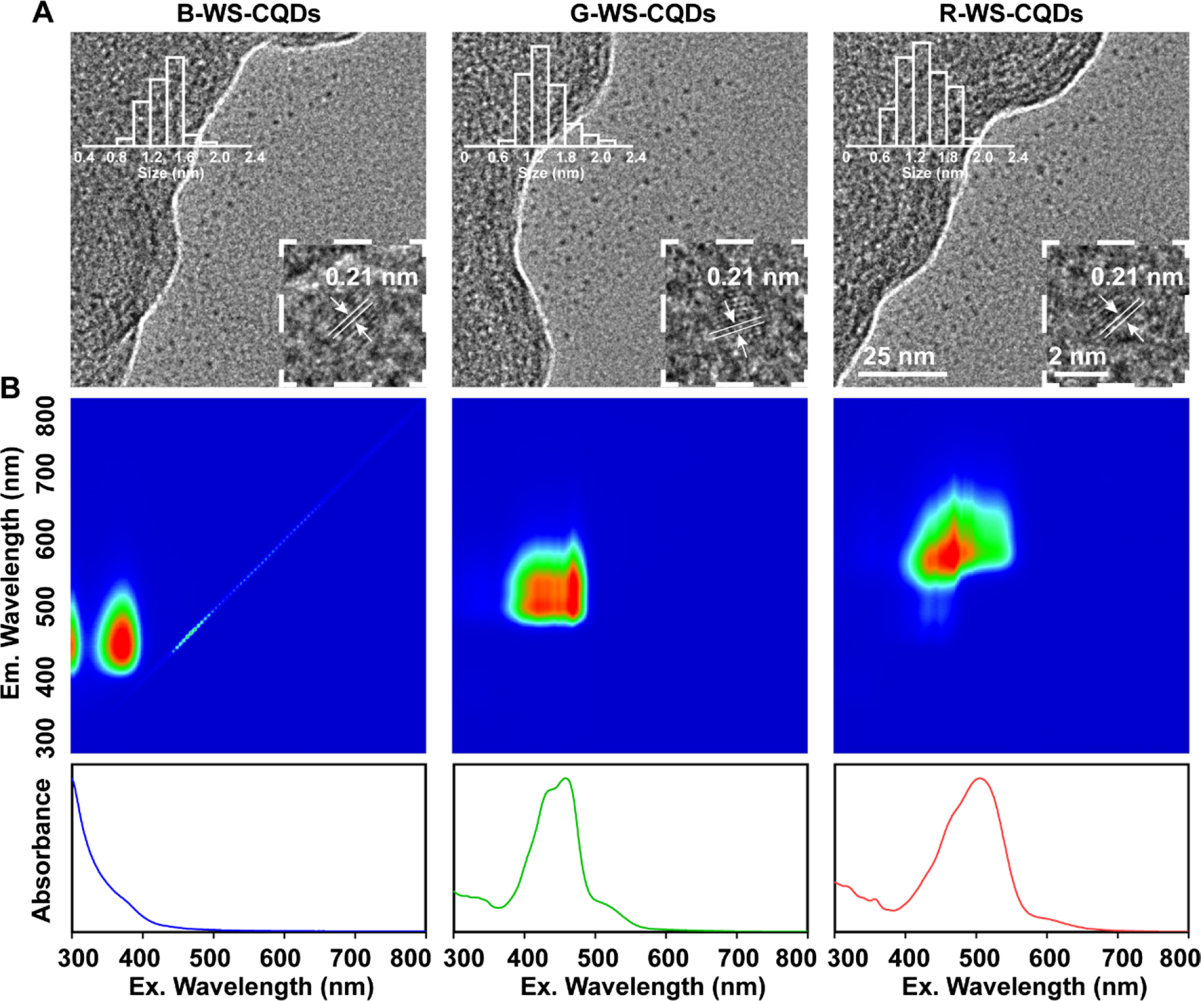
**A** TEM image of TC-WS-CQDs. The insets are size distribution of TC-WS-CQDs (upper left) and HRTEM image (lower right). **B** PL EEM (upper) and UV–Vis absorption (lower) of the TC-WS-CQDs under different excitation wavelengths. B-, G-, and R-WS-CQDs refer to Blue-, Green-, and Red-WS-CQDs respectively

TC-WS-CQDs were synthesized from an optimized proportion of tryptophan and sorbitol (100:500) in a one-pot hydrothermal method at 160 °C. The results showed that tryptophan had an important influence on the optical characteristics of the TC-WS-CQDs, and the addition of sorbitol could significantly reduce the proportion of amorphous carbon dots. The reactant was centrifuged to remove the precipitate, and the silica gel column chromatography was performed with ethanol and ethyl acetate as the eluent. In the process of column chromatography, rainbow-colored bands, mainly blue, green, and red, could be observed (Additional file 1: Figure S1). The B-, G-, and R-WS-CQDs carbon quantum dots were collected sequentially by gradient elution. TEM images showed that the B-, G-, and R-WS-CQDs had similar sizes and shapes with an average diameter of ≈ 1.4 nm (Fig. 1A). High-resolution TEM (HRTEM) images showed that all three types of TC-WS-CQDs exhibit clear lattice fringes with a spacing of 0.21 nm, which corresponds to the graphene (100) in-plane lattice, indicating that the difference in color of TC-WS-CQDs came from the surface and/or edges chemical composition rather than the size of the quantum dots (Fig. 1A inset). Compared with most of published synthetic methods of carbon dots, the synthesis of TC-WS-CQDs was simple, convenient and bio-friendly (Additional file 1: Table S1).

### Optical characterizations of TC-WS-CQDs

The most significant optical feature of B-, G- and R-WS-CQDs are continuous and different absorption and emission peaks. The fluorescence photoluminescence excitation-emission matrix (EEM) and the ultraviolet–visible (UV–Vis) absorption spectrum showed that the B-, G-, and R-WS-CQDs had strong absorption at the center wavelengths of 300 nm, 457 nm, and 540 nm, respectively, and emit fluorescence at the maximum emission wavelengths of 453 nm, 506 nm, and 581 nm (Fig. 1B), which allowed carbon quantum dots with different excitation wavelengths to be used separately in organisms.

### Chemical composition analysis

**Fig. 2:**
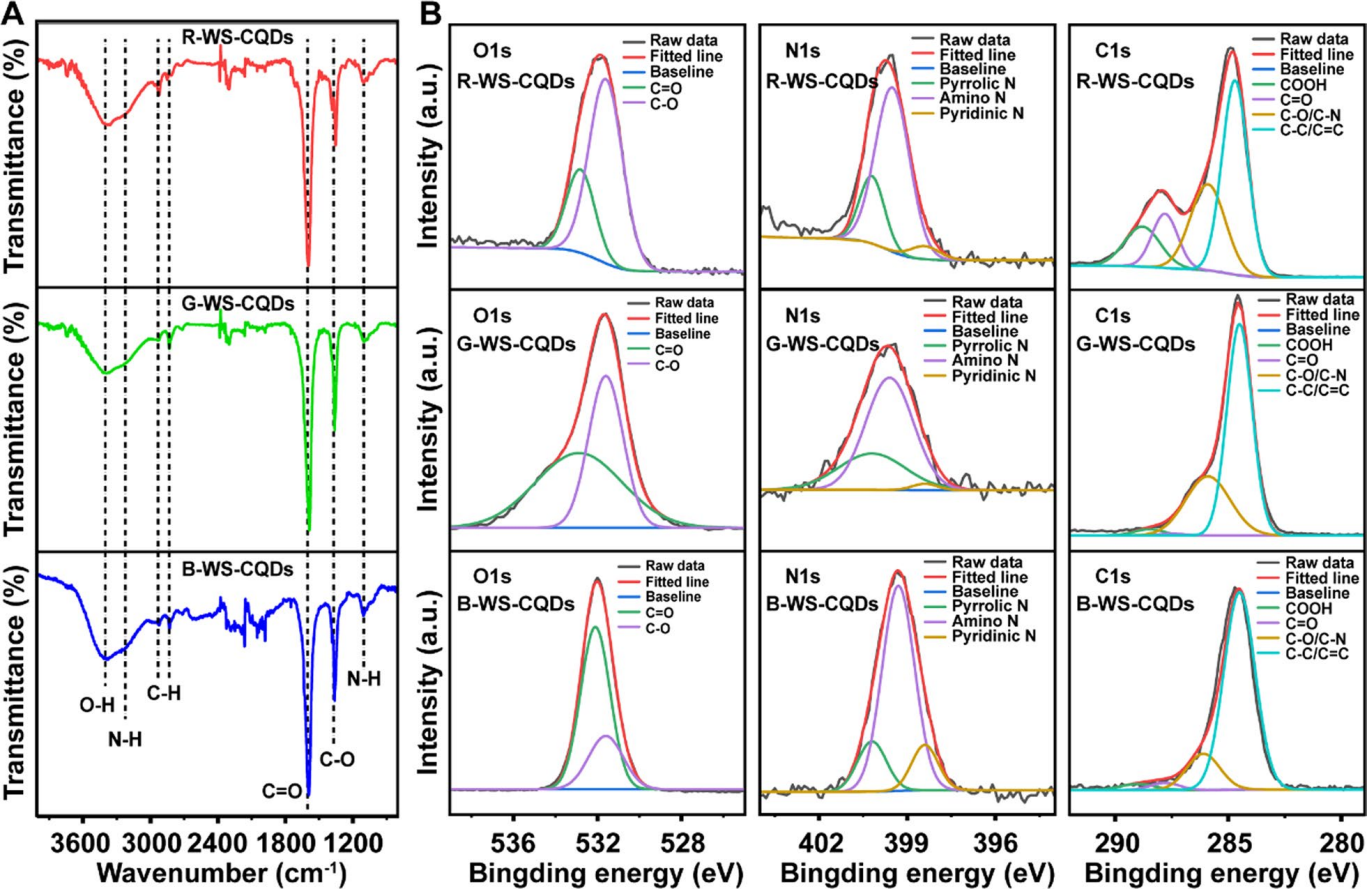
**A** FT-IR spectrum of B-WS-CQDs, G-WS-CQDs, and R-WS-CQDs. **B** High resolution X-ray photoelectron spectroscopy (XPS) spectrum of TC-WS-CQDs for O1s (left), N1s (medium), C1s (right)

In order to determine the mechanism of differences in optical properties of TC-WS-CQDs with the same size and crystal structure, fourier transform infrared (FT-IR) spectroscopy and X-ray photoelectron spectroscopy (XPS) were used for chemical composition analysis. The FT-IR spectrum (Fig. 2A) showed characteristic absorption peaks at 3395, 3212, 1592, and 1362 cm^−1^ were assigned to the stretching vibrations of O–H, N–H, C=O and C–O of B-, G- and R-WS-CQDs, suggesting that TC-WS-CQDs had similar groups. Furthermore, high-resolution XPS verified the FT-IR conclusion and confirmed the proportion of the groups. As shown in Fig. 2B, with the red shift of the emission wavelength, the proportion of hydroxyl and amino groups gradually increased, and the R-WS-CQDs contained a high proportion of amide bonds and carboxyl groups. The G-WS-CQDs had a higher proportion of pyrrolic N, indicating that G-WS-CQDs had a higher proportion of tryptophan residues.

### Cellular imaging and distribution of TC-WS-CQDs

**Fig. 3:**
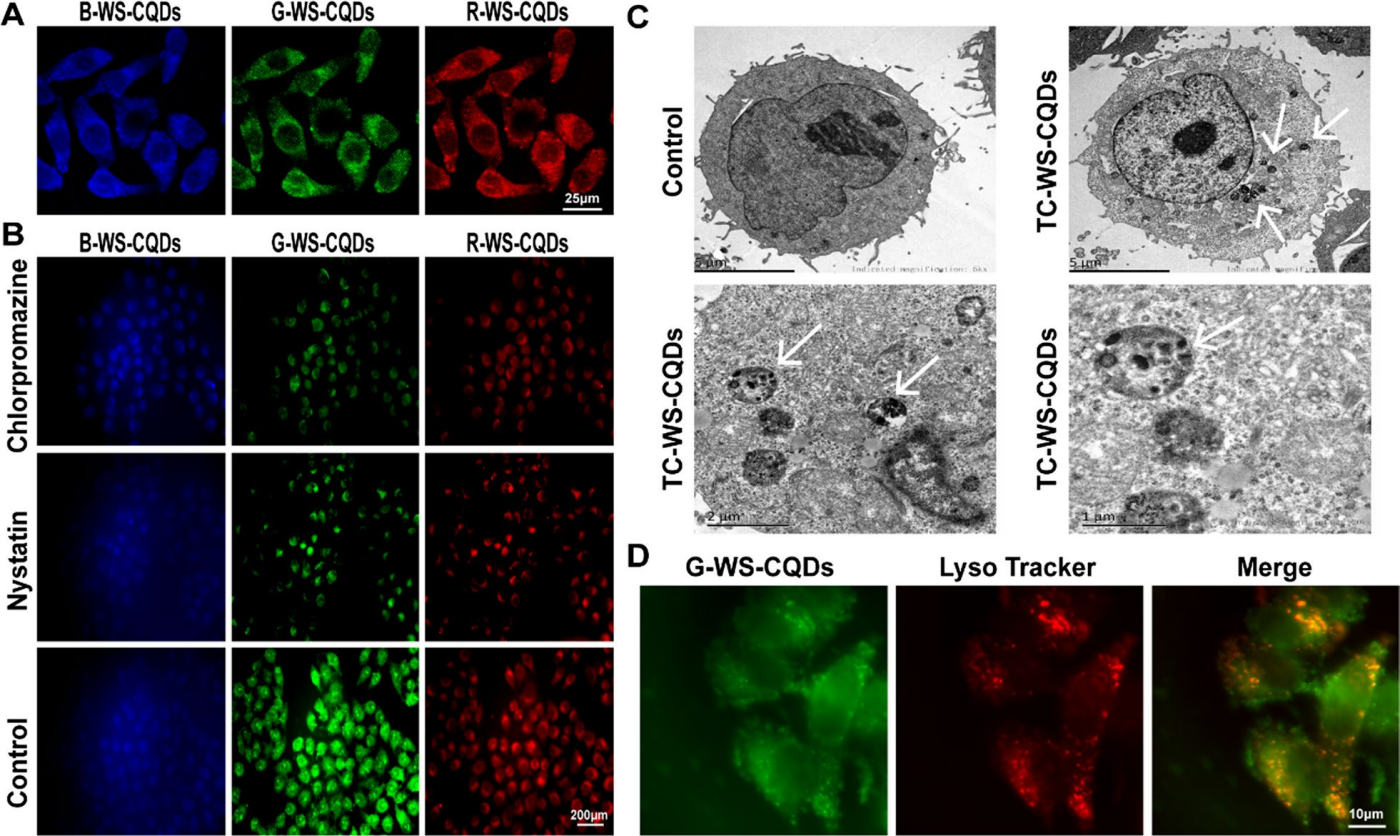
**A** LSCM images of Huh7 cells with TC-WS-CQDs incubation for 6 h. **B** Huh7 cells were treated with TC-WS-CQDs alone and co-treated with Nystatin (30 μM) or Chlorpromazine (20 μM) for 6 h. **C** The intracellular localization of TC-WS-CQDs was assessed by TEM in Huh7 cells. Arrows indicate intracellular vesicles engulfing TC-WS-CQDs in the cytoplasm. **D** G-WS-CQDs co-localization with lysosomes as tracked by LysoTracker

We detected the bioimaging of TC-WS-CQDs in Huh7 HCC cells. Using laser scanning confocal microscope (LSCM), B-, G-, and R-WS-CQDs generated blue, green, and red fluorescence respectively in Huh7 cells after incubation with TC-WS-CQDs for 6 h (Fig. 3A). To determine how TC-WS-CQDs entered Huh7 cells, we further monitored the endocytosis. After incubating with the endocytosis inhibitors (nystatin or chlorpromazine), the fluorescence intensity in Huh7 cells was decreased, indicating that the TC-WS-CQDs could enter cells by endocytosis (Fig. 3B). Additionally, TEM scanning revealed that intracellular localization of TC-WS-CQDs was mainly in the cytoplasm, and some were contained within intracytoplasmic vesicles including lysosome (Fig. 3C). LysoTracker Red further indicated most G-WS-CQDs were co-localization with lysosomes (Fig. 3D). These results confirm this carbon nanomaterial contains B-, G- and R-WS-CQDs and is mainly absorbed into lysosomes of the tumor cells in a proactive way.

In vivo distribution of TC-WS-CQDs, we quantified the fluorescence intensity of tissue homogenate using the microplate reader after injection of TC-WS-CQDs in C57BL/6J. As shown in Additional file 1: Figure S2, the fluorescence intensity was increased in the liver and in the kidney at 1 h after injection of TC-WS-CQDs, indicting TC-WS-CQDs could be enriched in the liver and kidney.

### Biotoxicity analysis of TC-WS-CQDs

**Fig. 4:**
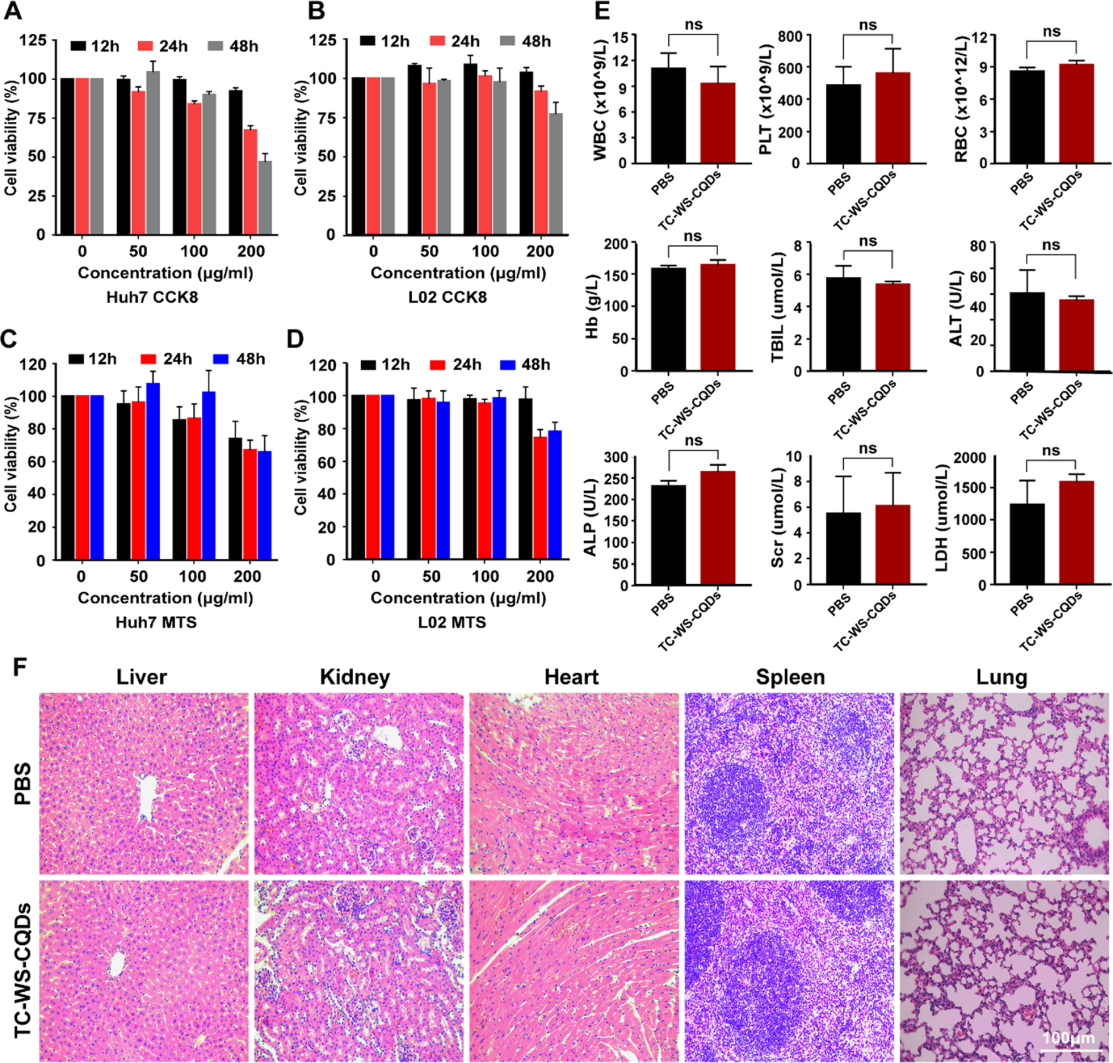
The biocompatibility of TC-WS-CQDs. **A**–**D** Cell viability of Huh7 cells and L02 cells, CCK8 assay (**A**, **B**), MTS assay (**C**, **D**). Cells were seeded to 96-well plates (8 × 10^3^ cells/well). **E**, **F** Toxicity assessment of TC-WS-CQDs in BALB/nu mice (n = 5). The mice were intravenously injected with TC-WS-CQDs or PBS (300 μl) every 2 days. The blood and organs were collected at day 14, **E** Complete blood count test and serum biochemistry results. **F** Histological evaluation of the major organs of the mice. *WBC* white blood cells, *PLT* platelet, *RBC* red blood cells, *Hb* hemoglobin, *TBIL* total bilirubin, *ALT* alanine aminotransferase, *ALP* alkaline phosphatase, *Scr* serum creatinine, *LDH* lactate dehydrogenase. Data represent mean ± SD (n = 3 in **A**–**D**, n = 5 in **E**, **F**, Mann–Whitney test or unpaired t test were used when appropriate for statistical significance analysis in **E**. ns: not significant)

The cytotoxicity of TC-WS-CQDs in Huh7 cells and L02 cells were evaluated by CCK8 and MTS assay. TC-WS-CQDs did not suppress Huh7 cells survival in a dose and time-dependent way until its concentration was up to 200 μg/ml (Fig. 4A and C), which was a higher concentration than that was needed for application. TC-WS-CQDs exhibited less toxicity in L02 cells (Fig. 4B and D). Meanwhile, TC-WS-CQDs had no obvious toxicity in BALB/nu mice mice after injecting TC-WS-CQDs for 14 days. As shown in Fig. 4E, TC-WS-CQDs did not cause abnormal serum index. Consistent with the serological test results, HE staining revealed no injury of organs after injecting TC-WS-CQDs (Fig. 4F). No toxicity was observed in C57BL/6J mice (Additional file 1: Figure S3).

For many years, semiconductor quantum dots are used as biosensors in medical studies, because of excellent fluorescence emission. However, their remarkable toxicity induced by heavy metals limits safe use for clinical applications [26]. Based on these results, TC-WS-CQDs have excellent biocompatibility with minimal toxicity, showing a promising nanomaterial for medical applications.

### TC-WS-CQDs selectively targeting hepatoma cells

To investigate the selectivity of TC-WS-CQDs towards hepatoma cells, Huh7 cells and L02 cells were incubated with TC-WS-CQDs (200 μg/ml) separately. Blue and red fluorescence between Huh7 cells and L02 cells were not obviously different. However, upon incubation with TC-WS-CQDs for 4 h, Huh7 cells showed bright green fluorescence at 470 nm excitation, while non-cancerous L02 cells appeared relatively faint green fluorescence (Additional file 1: Figure S4A). Over 6 h incubation with TC-WS-CQDs, the green fluorescence intensity of Huh7 cells enhanced rapidly and had significant differences compared with that of L02 cells (Additional file 1: Figure S4B–D). It is expected that the tumor cells might prefer to swallow G-WS-CQDs compared with normal hepatocytes, which implies a tumor targeting characteristics of the TC-WS-CQDs.

### TC-WS-CQDs for photodynamic therapy in vitro

**Fig. 5:**
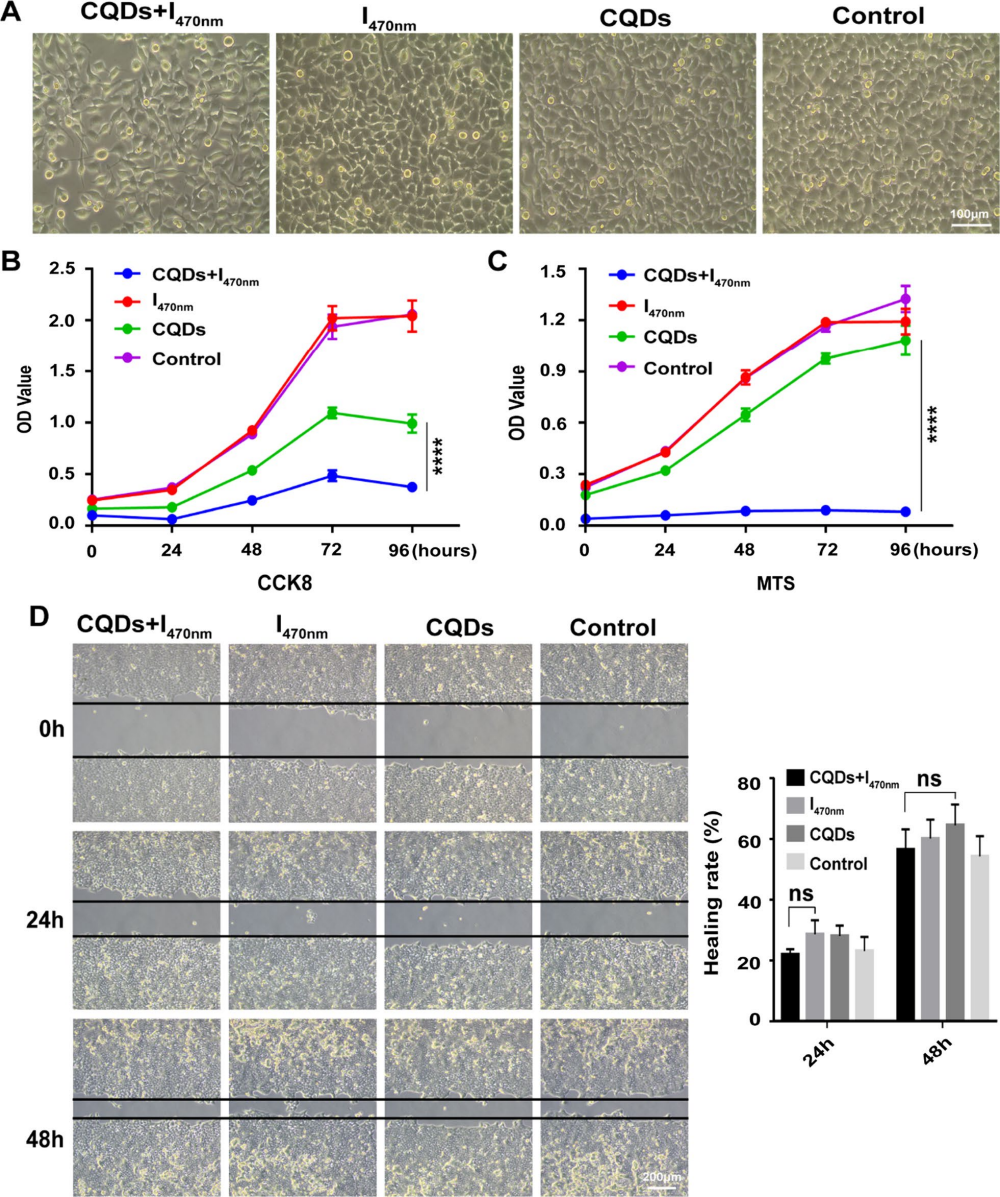
Photoexcited TC-WS-CQDs improved killing cells. Huh7 cells were treated with or without TC-WS-CQDs and exposed to 470 nm irradiation for 10 min. After 24 h incubation, the cell morphology was observed by optical microscope (**A**). The cell proliferation was assessed by CCK8 assay (**B**) and MTS assay (**C**). Microscope image (the left panel) and statistical analysis (the right panel) showed cell migration capacity by wound healing assay (**D**). The data are mean ± SD (n = 3, statistical significance was analyzed via two-way ANOVA in **B** and **C**, statistical significance was analyzed via Mann–Whitney test in **D**, ****p < 0.0001, ns: not significant). CQDs + I_470nm_: TC-WS-CQDs with 470 nm irradiation, I_470nm_: 470 nm irradiation without TC-WS-CQDs, CQDs: TC-WS-CQDs, Control: no TC-WS-CQDs and irradiation

To detect the potential efficiency of TC-WS-CQDs for PDT, we assessed the proliferation of Huh7 cells using the TC-WS-CQDs irradiated for 10 min with different wavelengths. The cells died upon irradiation at 365 nm without TC-WS-CQDs (Additional file 1: Figure S5A). In addition, TC-WS-CQDs with irradiation at 545 nm had no effects on proliferation of Huh7 cells (Additional file 1: Figure S5B, C). However, Huh7 cells were inhibited significantly upon irradiation at 470 nm (Fig. 5A–C), but no effects on cell migration were observed (Fig. 5D). These results indicated 470 nm photoexcited TC-WS-CQDs in PDT for hepatocellular carcinoma at the cellular level.

### TC-WS-CQDs activating autophagy via producing ROS

**Fig. 6:**
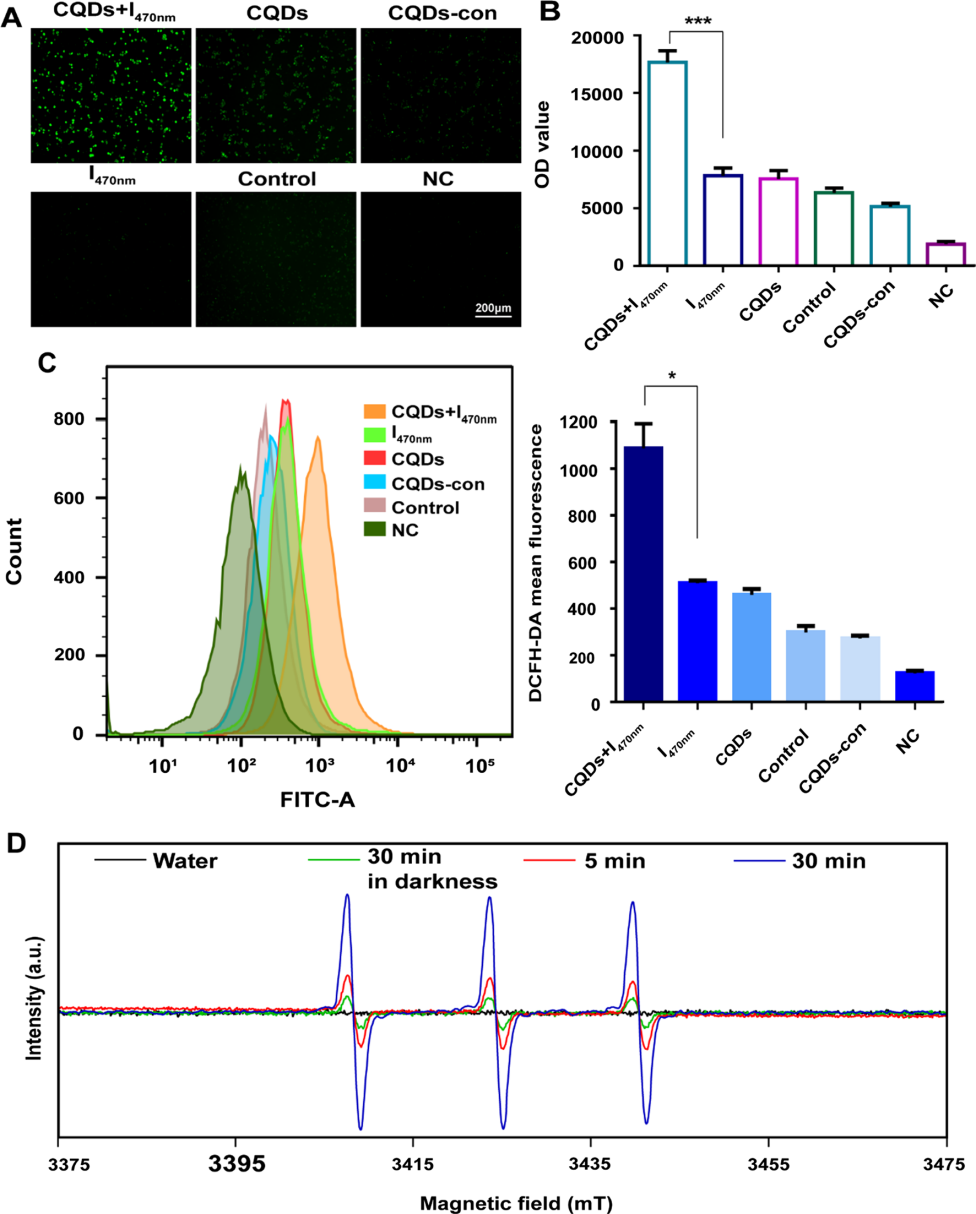
ROS generation by photoexcited TC-WS-CQDs. Huh7 cells were incubated with TC-WS-CQDs and exposed to 470 nm irradiation for 10 min. After 24 h, the cellular ROS was tested by DCFH-DA, fluorescence microscope measuring (**A**), microplate reader detection (**B**), flow cytometry (**C**). The data in **B**–**C** are mean ± SD (n = 3, statistical significance was analyzed via unpaired t test, *p < 0.05, ***p < 0.001). **D** ESR spectra of the TC-WS-CQDs in different irradiation time with 470 nm laser. (**A**–**C**, CQDs + I_470nm_: TC-WS-CQDs with 470 nm irradiation and DCFH-DA staining, I_470nm_: 470 nm irradiation and DCFH-DA staining without TC-WS-CQDs, CQDs: TC-WS-CQDs and DCFH-DA staining without irradiation, Control: DCFH-DA staining without TC-WS-CQDs and irradiation, CQDs-con: adding TC-WS-CQDs only without irradiation and DCFH-DA staining, NC: blank control, no TC-WS-CQDs, irradiation and DCFH-DA staining)

Considering photodynamic cytotoxicity of TC-WS-CQDs with 470 nm irradiation, we detected the intracellular ROS levels in Huh7 cells via DCFH-DA after incubation with TC-WS-CQDs. Consistent with our previous result of PDT in Huh7 cells, TC-WS-CQDs irradiation at 545 nm did not induce intracellular ROS generation (Additional file 1: Figure S6A–C). However, fluorescence imaging showed increasing ROS in Huh7 cells with the TC-WS-CQDs under irradiation at 470 nm (Fig. 6A). Quantitative analysis with microplate reader or flow cytometry also displayed a significant increase in intracellular ROS production of 470 nm irradiated TC-WS-CQDs (Fig. 6B and C). Moreover, the ROS inhibitors (NAC and APO) didn’t decrease ROS level (Additional file 1: Figure S7A, B), thus indicating that ROS sources were not mitochondrial-derived ROS and NADPH oxidase-induced ROS. Additionally, the ^1^O_2_-generation capability of the TC-WS-CQDs was investigated by ESR technique (Fig. 6D) with TEMP as an ^1^O_2_ trapper. These results proved that the ROS was exogenous, which generated from TC-WS-CQDs with 470 nm irradiation.

**Fig. 7:**
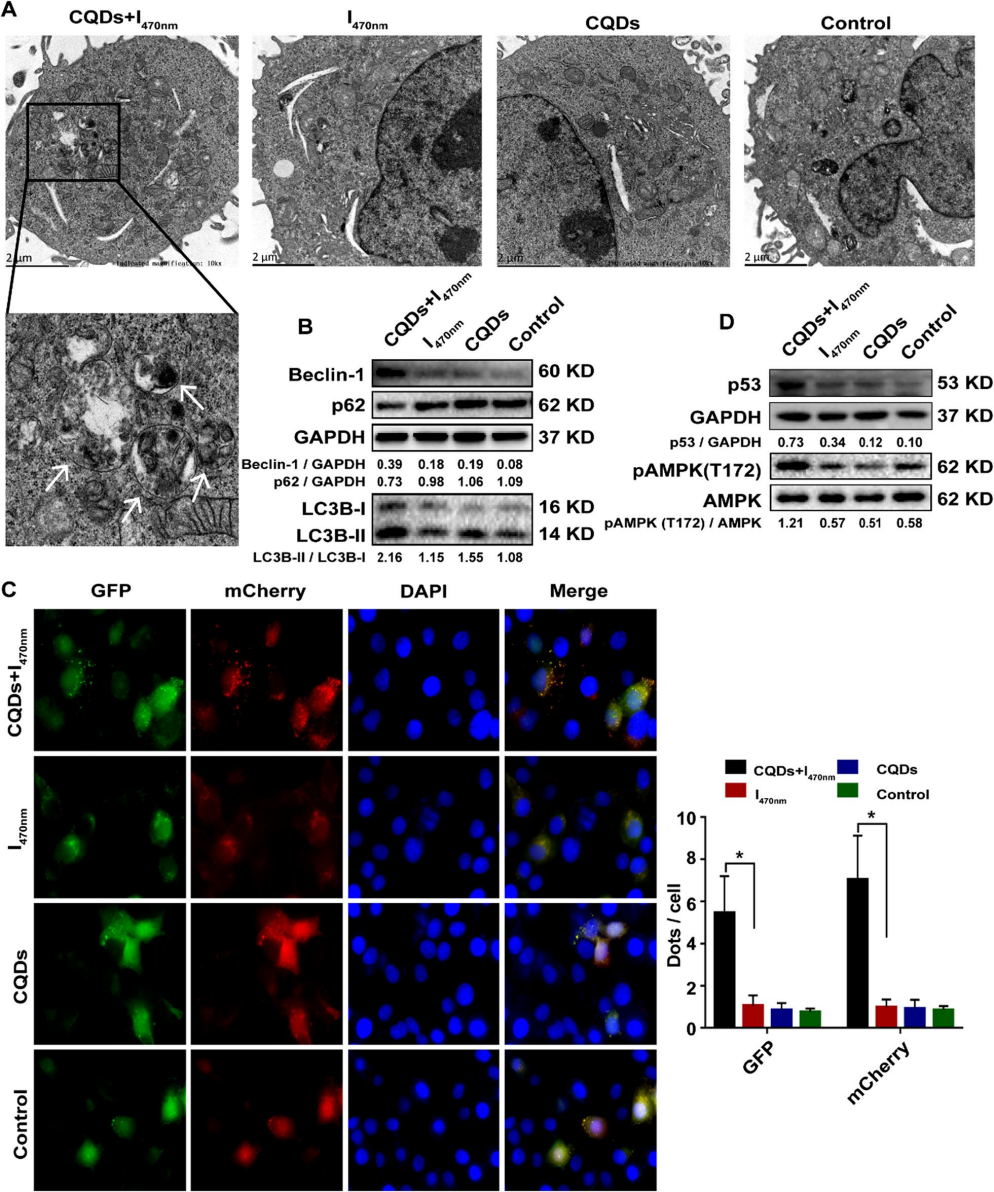
Induction of autophagy by photoexcited TC-WS-CQDs in Huh7 cells. **A** Detection of autophagosomes by TEM. Arrows indicate autophagosomes. **B** Western-blot analysis of LC3 conversion, expression of Beclin-1 and p62. **C** Fluorescence images of LC3 puncta expressing mcherry-GFP-LC3. Autophagosomes and autolysosomes were observed in Huh7 cells. The data in **C** are mean ± SD (n = 3, statistical significance was analyzed via Mann–Whitney test, *p < 0.05). **D** Western-blot analysis of expression of p53 and phosphorylation AMPK. CQDs + I_470nm_: TC-WS-CQDs with 470 nm irradiation, I_470nm_: 470 nm irradiation without TC-WS-CQDs, CQDs: TC-WS-CQDs, Control: no TC-WS-CQDs and irradiation

As shown in Fig. 3C and D, lots of the TC-WS-CQDs entered lysosomes in tumor cells, which may represent occurrence of autophagy caused by ROS [27]. Given that TC-WS-CQDs generated ROS upon photoexcitation at 470 nm, we further assessed whether TC-WS-CQDs caused autophagy in tumor cells. TEM analysis showed the generation of autophagosomes in 470 nm-irradiated TC-WS-CQDs group (Fig. 7A). Immunoblot assay revealed the conversion of LC3B-I to LC3B-II, upgradation of Beclin-1 and degradation of p62 under 470 nm-irradiated TC-WS-CQDs, which indicated the formation of autophagosomes and autolysosomes (Fig. 7B). mCherry-GFP-LC3 fluorescence microscope assay confirmed the increasing numbers of autophagosomes and autolysosomes by 470 nm-irradiated TC-WS-CQDs (Fig. 7C). Additionally, flow cytometry also indicated a larger LC3B signal in 470 nm-irradiated TC-WS-CQDs group (Additional file 1: Figure S8). Besides, immunoblot assay showed that p53 and pAMPK upgraded in 470 nm-irradiated TC-WS-CQDs group (Fig. 7D), indicating p53 signaling as a mediator of the autophagic pathway in Huh7 cells. However, apoptotic parameters had no significant changes in Huh7 cells with 470 nm-irradiated and non-irradiated TC-WS-CQDs (Additional file 1: Figure S9).

### TC-WS-CQDs for photodynamic therapy in vivo

**Fig. 8:**
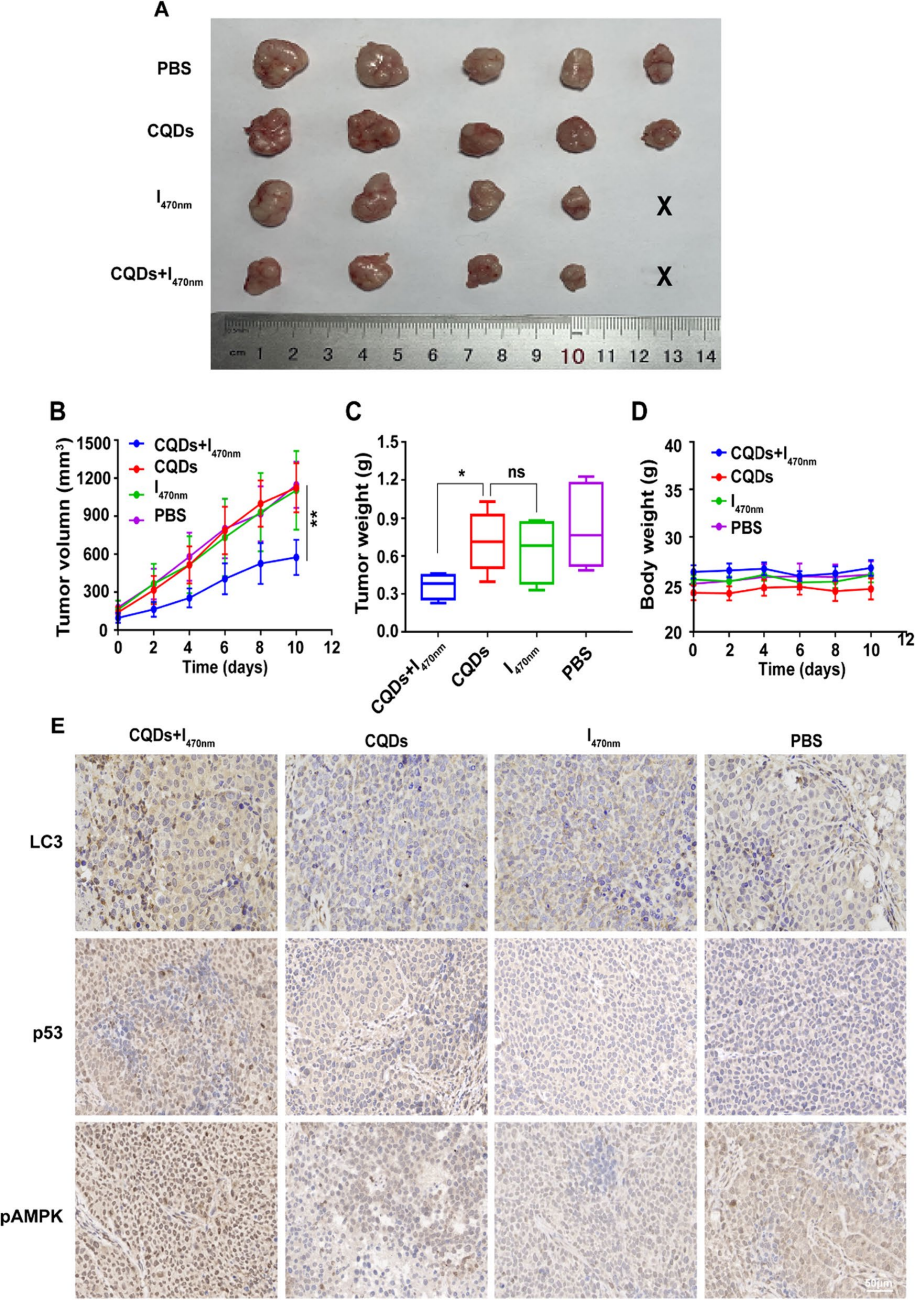
In vivo PDT of TC-WS-CQDs. **A** Photos of the excised tumors on day 10 (X: failed to form tumor). **B** The tumor volume of mice as a function of time. **C** Quantitative analysis of tumor weight on day 10. **D** Body weight of Huh7 cancer bearing mice of different groups after treatments. **E** The detection of LC3, p53 and pAMPK expression in tumors by immunohistochemistry. The data are mean ± SD (n = 5, statistical significance was analyzed via two-way ANOVA in **B**, statistical significance was analyzed via unpaired t test in **C**, *p < 0.05, **p < 0.01, ns: not significant. CQDs + I_470nm_: TC-WS-CQDs with 470 nm irradiation, I_470nm_: 470 nm irradiation without TC-WS-CQDs, CQDs: TC-WS-CQDs, PBS: no TC-WS-CQDs and irradiation

To address whether the TC-WS-CQDs exerted potential therapeutic role against HCC in vivo, we next performed animal PDT experiments. Mice were intratumorally injected with PBS or TC-WS-CQDs, and the tumor site was subjected to 470 nm irradiation or no irradiation. The body weight of mice and the tumor volume were measured every 2 days. After the treatment for 10 days, all mice were sacrificed and the tumors were excised and weighed. The results showed that the tumor size was the smallest in CQDs + I_470nm_ group (Fig. 8A). Both the tumor volume and the tumor weight in CQDs + I_470nm_ group had significant differences compared with other groups (Fig. 8B and C). Meanwhile, the body weight had no significant difference between the four groups during the experiment (Fig. 8D). Immunohistochemistry revealed LC3, p53 and pAMPK were high expression in CQDs + I_470nm_ group (Fig. 8E). Collectively, PDT of TC-WS-CQDs for HCC was further validated in vivo.

Currently, sorafenib and lenvatinib are the first-line approved agents in HCC [28, 29]. Although sorafenib can prolong survival in HCC patients, the response rate is usually low and its efficacy is short owing to the development of resistance [28, 30]. In addition, traditional chemotherapeutic drugs often cause systemic toxicity because they are not well-targeted to the tumors. TC-WS-CQDs, targeting to HCC cells, showed an effective anti-HCC efficacy without toxicity. Herein, we demonstrated a novel strategy for HCC treatment with no systemic toxicities compared to the popular chemotherapeutic drugs.

## Conclusions

In summary, we have developed a theranostic nanoplatform of TC-WS-CQDs via environmental-friendly hydrothermal decomposition of tryptophan and sorbitol for HCC treatment. In contrast to normal hepatocyte, higher targeting into HCC cells by the TC-WS-CQDs may lead to early monitoring of tumor cells through fluorescence imaging. Simultaneously, the green-emitting TC-WS-CQDs enhance HCC prevention in vitro and in vivo without any drug-delivery. This anticancer therapy is due to ROS generation activating autophagy via p53-AMPK pathway. This study may highlight a promising anticancer nanotheranostic strategy with integration of diagnosis, targeting, and therapy.

## Supplementary Information

**Additional file 1.** Supplementary table and figures.

## Data Availability

All data generated or analyzed during this study are included in this published article and its additional information files.

## Abbreviations

HCC: Hepatocellular carcinoma
PD-1: Programmed cell death protein 1
PD-L1: Programmed cell death-ligand protein 1
CQDs: Carbon quantum dots
PL: Photoluminescence
TC-WS-CQDs: Trichrome-tryptophan-sorbitol CQDs
ROS: Reactive oxygen species
DMEM: Dulbecco’s Modified Eagle’s Medium
RPMI: Roswell Park Memorial Institute
FBS: Fetal bovine serum
CCK8: Cell Counting Kit-8
MTS: 3-(4,5-Dimethylthiazol-2-yl)-5-(3-carboxymethoxyphenyl)-2-(4-sulfophenyl)-2H-tetrazolium, inner salt
PBS: Phosphate-buffered saline
NAC: *N*-Acetylcysteine
APO: Apocynin
^1^O_2_: Singlet oxygen
OD: Optical density
DCFH-DA: 2′,7′-Dichlorodi-hydrofluorescein diacetate dye
TEMP: 2,2,6,6-Tetramethylpiperidine
ESR: Electron spin resonance
HE: Hematoxylin–eosin
PDT: Photodynamic therapy
TEM: Transmission electron microscopy
HRTEM: High-resolution TEM
EEM: Excitation-emission matrix
UV–Vis: Ultraviolet–visible
FT-IR: Fourier transform infrared
XPS: X-ray photoelectron spectroscopy
LSCM: Laser scanning confocal microscope
WBC: White blood cells
PLT: Platelet
RBC: Red blood cells
Hb: Hemoglobin
TBIL: Total bilirubin
ALT: Alanine aminotransferase
ALP: Alkaline phosphatase
Scr: Serum creatinine
LDH: Lactate dehydrogenase
