# Supplementary material for: Tryptophan-sorbitol based carbon quantum dots for theranostics against hepatocellular carcinoma
Source: J Nanobiotechnology. 2022 Feb 14;20:78. doi: 10.1186/s12951-022-01275-2 (PMC8842979; doi:10.1186/s12951-022-01275-2)
Supplement: Supplementary file 1 — Additional file 1. Supplementary table and figures. [file 12951_2022_1275_MOESM1_ESM.docx]

Additional Information

**Tryptophan-Sorbitol Based Carbon Quantum Dots for Theranostics against** **Hepatocellular Carcinoma**

Yang Wang^#^, Jun Chen^#^, Jiekang Tian^#^, Guanchen Wang, Weikang Luo, Zebing Huang, Yan Huang, Ning Li*, Mingming Guo*, Xuegong Fan*

^#^ These authors contributed to this work equally.

*Corresponding authors: Xuegong Fan: xgfan@hotmail.com; MingMing Guo: guomm57@swu.edu.cn; Ning Li: liningxy@csu.edu.cn

**Experimental section**

Cell culture and reagents

The liver cancer cell line Huh7 and the normal liver cell line L02 were obtained from Key Laboratory of Viral Hepatitis of Hunan Province. Huh7 cells and L02 cells were cultured in DMEM media and RIPA media, respectively, with 10% fetal bovine serum (FBS) and 1% penicillin/streptomycin supplementation. Cells incubated at 37℃ in a humidified incubator containing 5% CO_2_.

Distribution of TC-WS-CQDs in vivo

For the in vivo distribution, C57BL/6J male mice (7 weeks old) were purchased from Hunan SJA Laboratory Animal Company. The mice were treated with injection of 300 ul TC-WS-CQDs through tail vein. The mice were sacrificed and the organs were collected at 0h, 1h, 2h, 4h and 6h after injection respectively. The organs were grinded using a tissue grinder (SCIENTZ-48). Then the tissue homogenate was treated with an ultrasonic pulverizer (SCIENTZ08-III). The tissue homogenate was subsequently centrifuged at 12,000 rpm for 10 min and the supernatants were collected. The fluorescence intensity was quantified by a microplate reader under the excitation wavelength of 405 nm and emission wavelength of 445 nm.

Fluorescence intensity detection in Huh7 cells and L02 cells

Huh7 cells and L02 cells were incubated with TC-WS-CQDs (100 μg/ml) separately in 24-well plates with cell climbing slice or 12 wells. After incubation for 1 h, 2 h, 4 h and 6 h, the cells were washed with PBS three times. Then the cell climbing slices fixed with 4% paraformaldehyde for fluorescence microscopy detection and the cells in 12 wells were collected for fluorescence intensity detection by a microplate reader.

LC3B detection in Huh7 cells by flow cytometry

Huh7 cells with different treatment were collected after incubation for 24 h and fixed with 2% paraformaldehyde for 15 min at room temperature (RT). Cells were then washed with PBS and permeabilized with 0.2% Triton X-100 for 20 min. Cells were incubated with 5 ug/ml anti-LC3B (L7543, Sigma) at RT for 45 min. Cells were washed with PBS and stanined with Goat anti-Rabbit IgG Cross-Adsorbed Secondary Antibody Alexa Fluor 488 (A-11008, Invitrogen) for 30 min at RT. After staining the cells, 100 μl cell suspensions in PBS were analyzed by flow cytometry.

Annexin V/propidium iodide staining

Huh7 cells with different treatment were seeded into 12-well plates. After 24 h of incubation, cells were harvested for double staining with Annexin V-fluorescein isothiocyanate (FITC) and propidium iodide. Staining was performed according to the manufacturer’s instructions (BD Pharmingen, US, 559763). After staining the cells, 100 μl cell suspensions in PBS were analyzed by flow cytometry immediately.

TUNEL staining

Apoptosis was also analyzed by using the TUNEL BrightRed Apoptosis Detection Kit (Vazyme, A311, China), following the supplier’s instructions. Huh7 cells cultured on cell climbing slices were washed with PBS and fixed with 4% paraformaldehyde. The fixed and permeabilized cells were incubated with terminal deoxynucleotidyl transferase (TdT) reaction reagent containing biotin dUTP. After 1 h of incubation at 37℃, the cells were washed three times. Then, avidin–fluorescein isothiocyanate (FITC) was added and the mixture was incubated for 30 min at room temperature. The stained cells were observed using fluorescence microscopy (Zeiss, SCOPE A1, Germany).

Instrumentation

The transmission electron microscopy (TEM) and high-resolution TEM (HRTEM) images of the B-, G-, R-WS-CQDs were obtained with a FEI Talos F200X operated at 200 kV. UV-Vis and FT-IR absorption spectra were measured by SHIMADZU UV-2600 and PerkinElmer FRONTIER spectrophotometer respectively. PL spectra were collected on the HORIBA Duetta fluorescence and absorbance spectrometer. XPS were performed on a Thermo Scientific Escalab 250Xi spectrometer. EPR spectra were recorded with a Bruker EMX nano spectrometer using a microwave power of 0.3162 mW.

Cell images were acquired with a Leica laser scanning confocal microscopy or a Zeiss fluorescence microscope. Optical microscope images were obtained with the Leica microscope system. The optical density and fluorescence intensity were measured by the PerkinElmer’s Ensight Multilabel Plate Reader. The western blots were detected using the BIO-RAD, ChemiDoc XRS+ enhanced chemiluminescence system.

**Additional table**

Table S1. Summary of recent advances in synthetic methods for CQDs

| Precursor | Syntetic method | PL emission (nm) | Biocompatibility | Application | Refs. |
| --- | --- | --- | --- | --- | --- |
| Urea and p-phenylenediamine | Hydrothermal treatment at 160 ℃ for 10 hours | 440 to 625 | Toxic precursor | Bioimaging | Ding et al. (2016) ^[1]^ |
| Polythiophene phenylpropionic acid | Hydrothermal treatment at 240 ℃ for 36 hours | 640 | Toxic precursor | Bioimaging, photoacoustic, and thermal theranostics | Ge et al. (2015)^[2]^ |
| Citric acid, urea, octadecylamine, mPEG-NH2, PEG-b-PPG-b-PEG, et al. | Solvothermal treatment at 160℃ and nanoprecipitation | 642 | Toxic precursor | ROS bioimaging | Shen et al. (2020)^[3]^ |
| Curcumin and folic acid | Hydrothermal treatment at 240 ℃ for 4 hours | 450 to 560 | Bio-friendly precursor | photodynamic therapy for oral cancer | Nasrin et al (2015)^[4]^ |
| Glucose and asparic acid | Hydrothermal treatment at 200 ℃ for 20 minutes | 475 to 635 | Bio-friendly precursor | Diagnosis for brain cancer | Zheng et al (2015)^[5]^ |
| Sorbitol and Tryptophan | Hydrothermal treatment at 160 ℃ for 10 hours | 453, 506, and 581 | Bio-friendly precursor | Diagnosis and treatment for live cancer | This work |

PL: Photoluminescence; ROS: Reactive Oxygen Species.

**Additional figures**


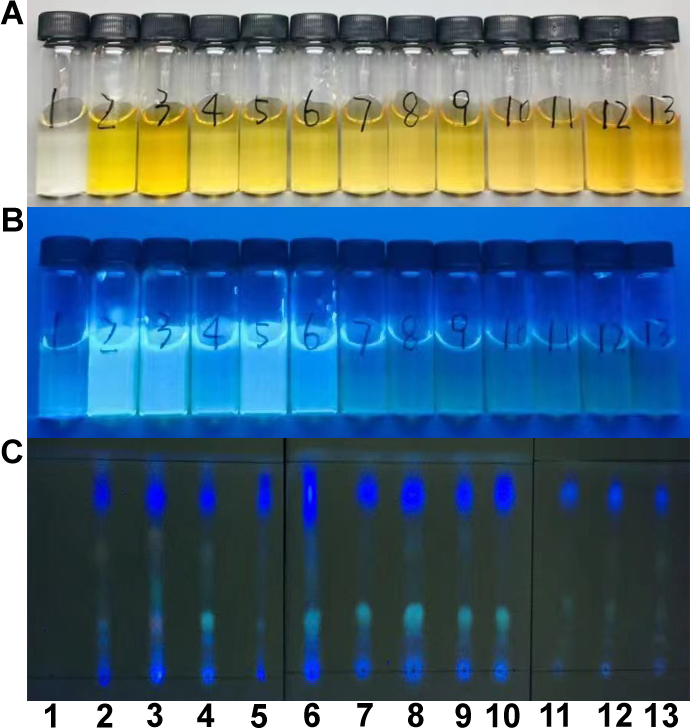


Figure S1. Photograph of the supernatant of the prepared TC-WS-CQDs after centrifugation under daylight (A) and 365 nm UV light (B), and thin layer chromatography (ethyl acetate: ethanol = 1:1 as developing solvent) results (C) under 365 nm UV light. From 1 to 13, these samples were produced using mass ratios of tryptophan to sorbitol of 0:600, 50:550, 100:500, 150:450, 200:400, 250:350, 300:300, 350:250, 400:200, 450:150, 500:100, 550:50, 600:0, respectively.


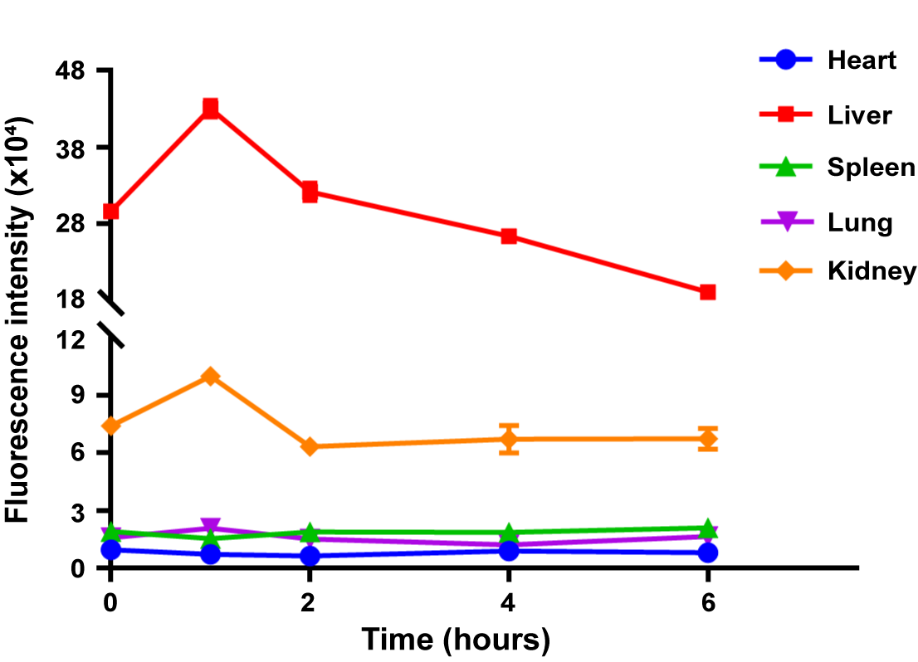


Figure S2. The biodistribution of TC-WS-CQDs in C57BL/6J mice. The mice were intravenously injected with TC-WS-CQDs (300 μl) and the organ fluorescence intensity after injection of different time was quantified by microplate reader under the excitation wavelength of 405 nm and emission wavelength of 445 nm. Data are mean ± SD, n = 3.


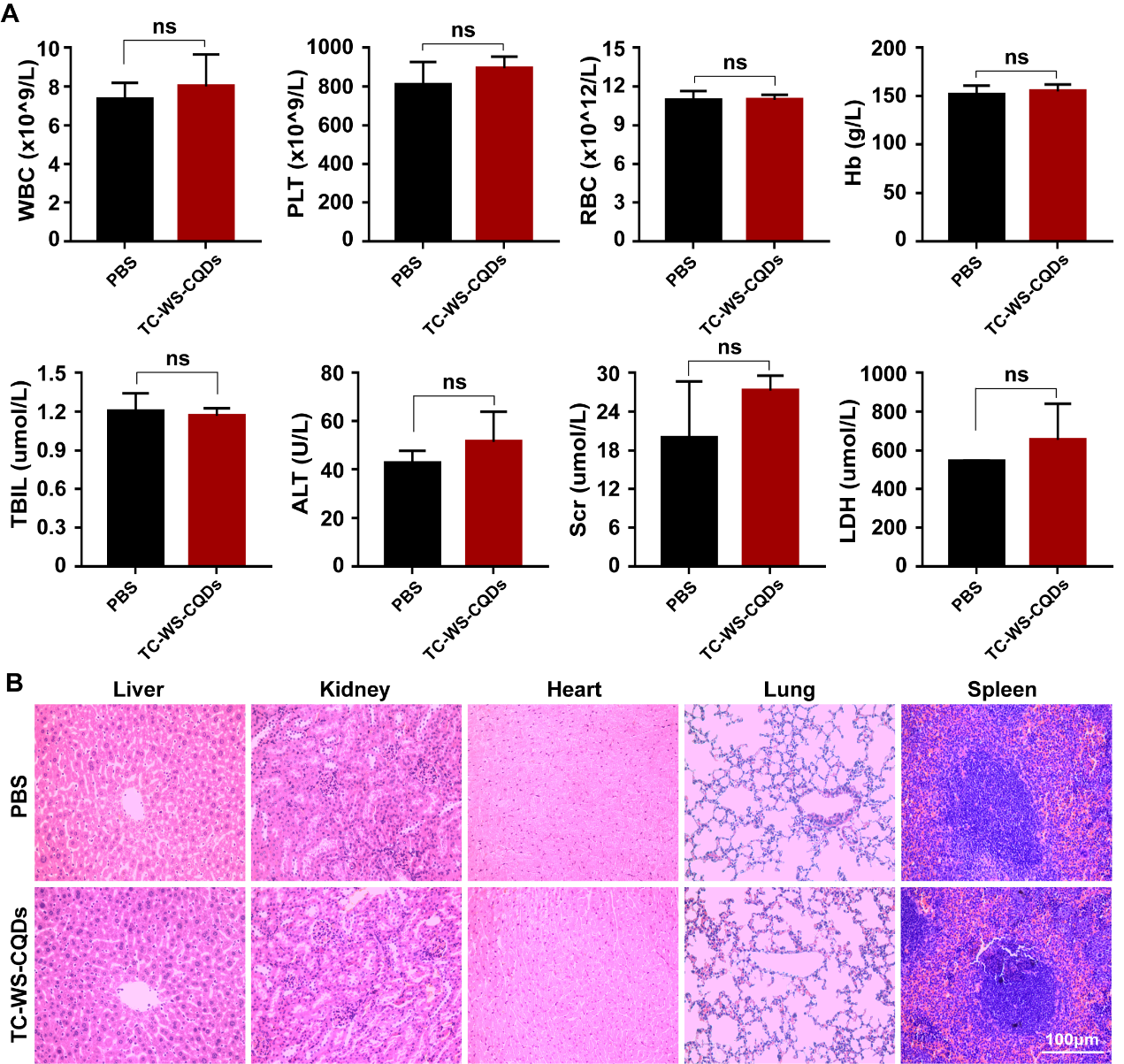


Figure S3. Toxicity assessment of TC-WS-CQDs in C57BL/6J mice. the mice were intravenously injected with TC-WS-CQDs or PBS (300 μl) every two days. The blood and organs were collected at day 14, (A) complete blood count test and serum biochemistry results. (B) histological evaluation of the major organs of the mice. WBC: white blood cells, PLT: platelet, RBC: red blood cells, Hb: hemoglobin, TBIL: total bilirubin, ALT: alanine aminotransferase, Scr: serum creatinine, LDH: lactate dehydrogenase. Data represent mean±SD (n = 3, Mann-Whitney test or unpaired t test were used when appropriate for statistical significance analysis, ns: not significant).


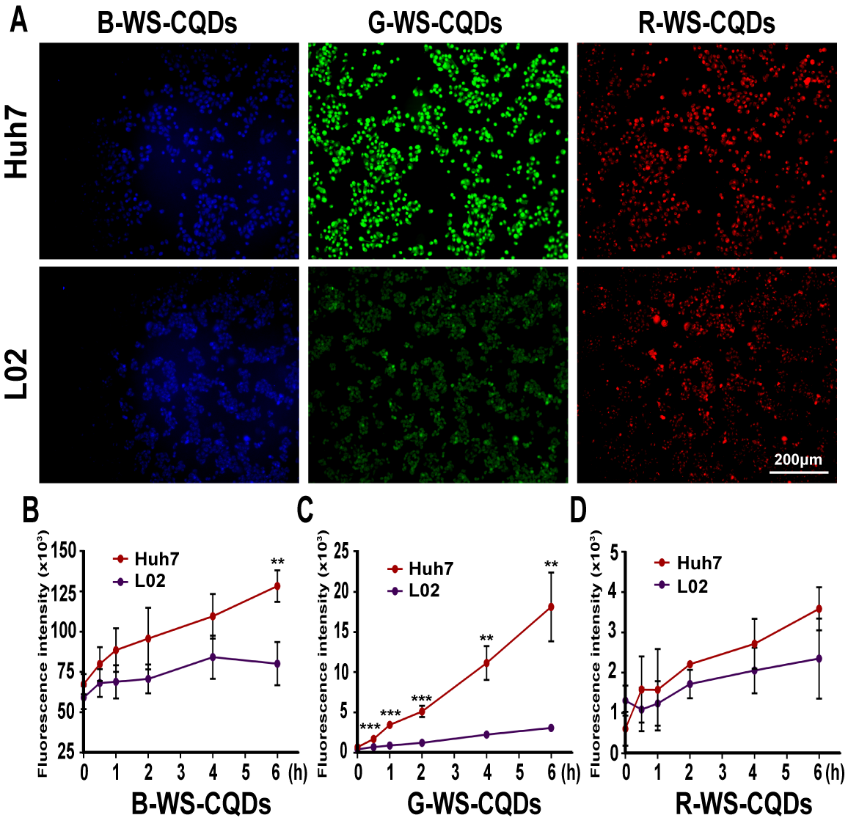


Figure S4. (A) Florescence microscope image of Huh7 cells and L02 cells after incubation with TC-WS-CQDs for 4 hours. (B-D) Microplate reader detecting the fluorescence intensity of Huh7 cells and L02 cells after incubation with TC-WS-CQDs, λ_365nm_ (B), λ_470nm_ (C), λ_545nm_ (D). Data in B-D are mean±SD (n= 3, Mann-Whitney test or unpaired t test were used when appropriate for statistical significance analysis.**p < 0.01, ***P < 0.001).


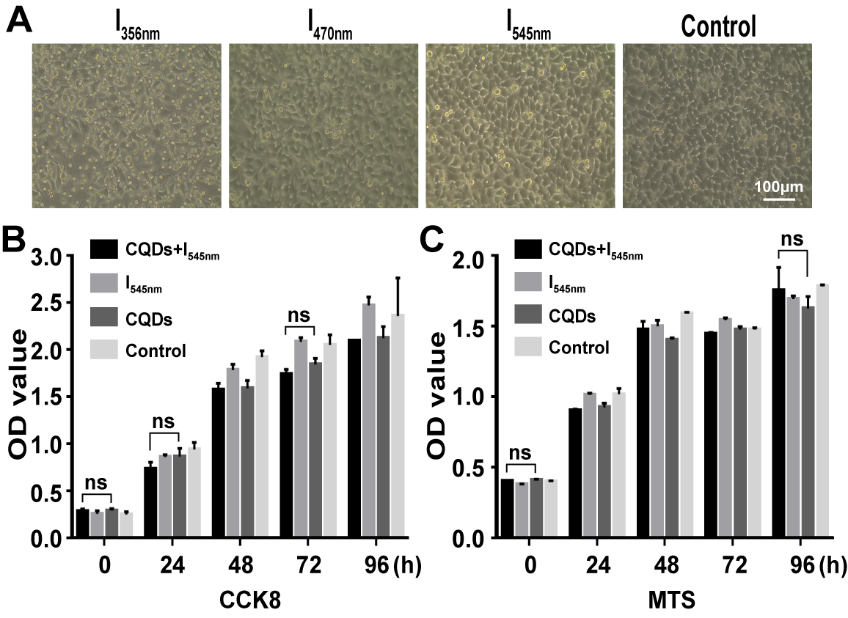


Figure S5. (A) Optical microscope analysis of cell morphology. Huh7 cells were treated with 356 nm, 470 nm, and 545 nm irradiation for 10 min. (B, C) Huh7 cells were treated with TC-WS-CQDs and exposed to 545 nm irradiation for 10min. The cell proliferation was assessed by CCK8 assay and MTS assay, respectively. Data in B,C are mean±SD (n = 3, Mann-Whitney test or unpaired t test were used when appropriate for statistical significance analysis, ns: not significant). CQDs+I_545nm_: TC-WS-CQDs with 545 nm irradiation, I_545nm_: 545 nm irradiation without TC-WS-CQDs, CQDs: TC-WS-CQDs, Control: no TC-WS-CQDs and irradiation.


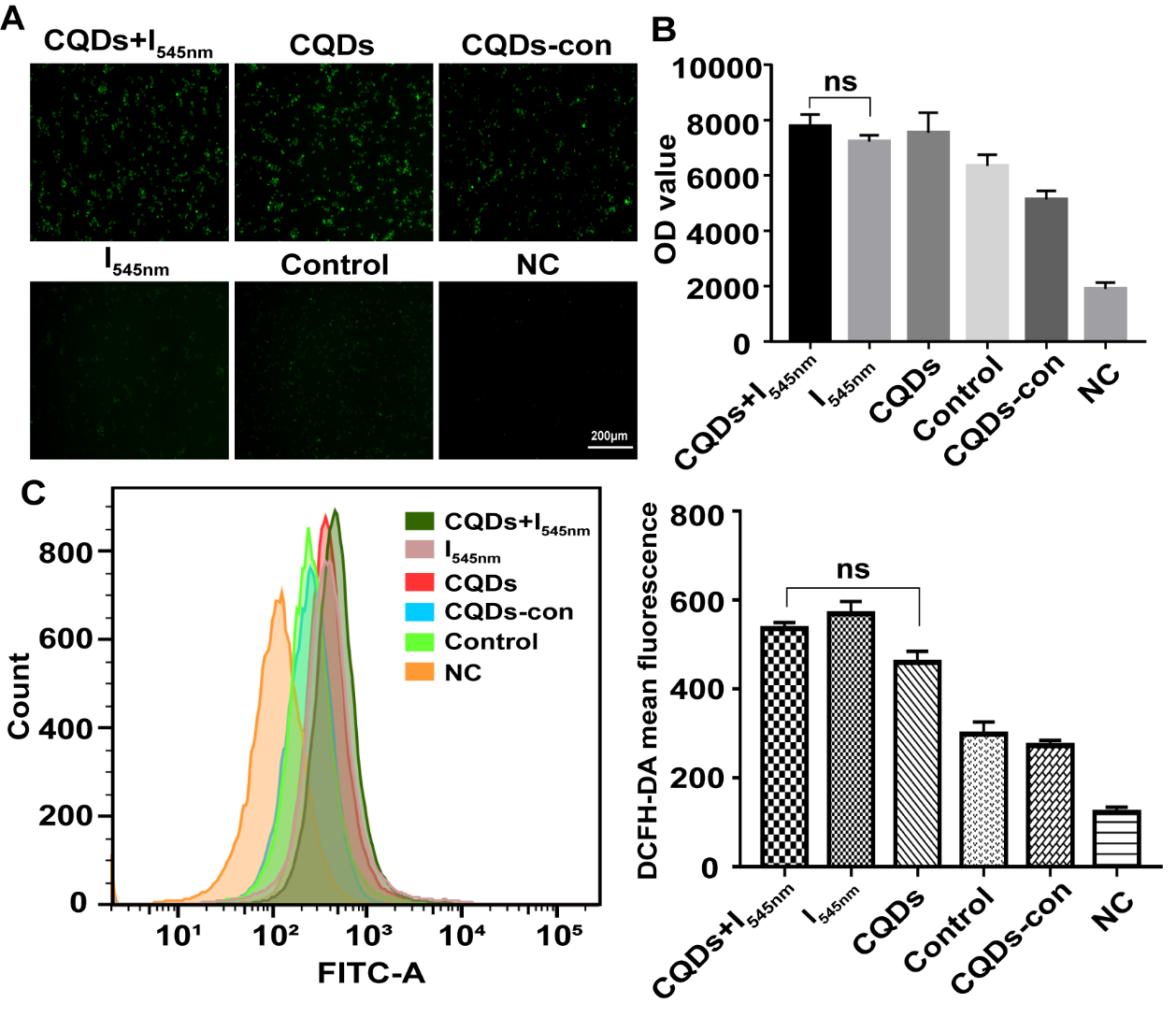


Figure S6. Cellular ROS levels by 545 nm photoexcited TC-WS-CQDs. Huh7 cells were incubated with TC-WS-CQDs and exposed to 545 nm irradiation for 10 min. After 24h, the cellular ROS was tested by DCFH-DA, fluorescence microscope measuring (A), microplate reader detection (B), flow cytometry (C). The data in B and C are mean±SD (n = 3, Mann-Whitney test was used for statistical significance analysis, ns: not significant). CQDs+I_545nm_: TC-WS-CQDs with 545 nm irradiation and DCFH-DA staining, I_545nm_: 545 nm irradiation and DCFH-DA staining without TC-WS-CQDs, CQDs: TC-WS-CQDs and DCFH-DA staining without irradiation, Control: DCFH-DA staining without TC-WS-CQDs and irradiation, CQDs-con: adding TC-WS-CQDs only without irradiation and DCFH-DA staining, NC: blank control, no TC-WS-CQDs, irradiation and DCFH-DA staining.


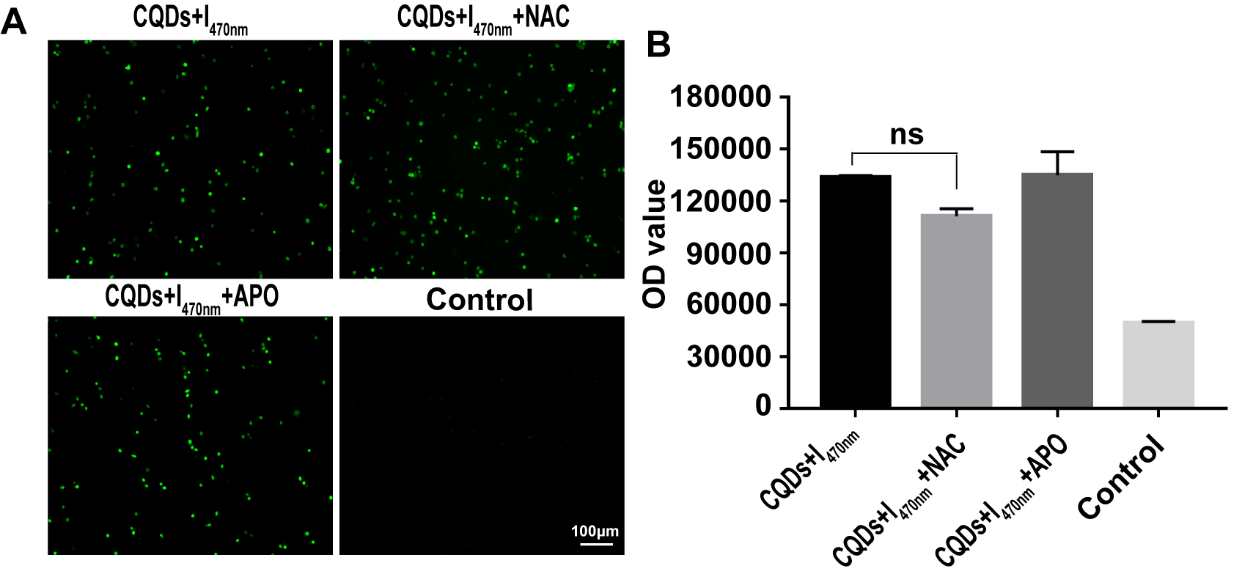


Figure S7. The effects of ROS inhibitors in inhibiting ROS levels. Huh7 cells were treated with 470 nm photoexcited TC-WS-CQDs + NAC (5 mM) or 470 nm photoexcited TC-WS-CQDs + APO (2 mM). After 24 h, ROS levels were tested by DCFH-DA. (A) fluorescence microscope measuring, (B) microplate reader detection. Data in B are mean±SD (n = 3, Mann-Whitney test was used for statistical significance analysis, ns: not significant).


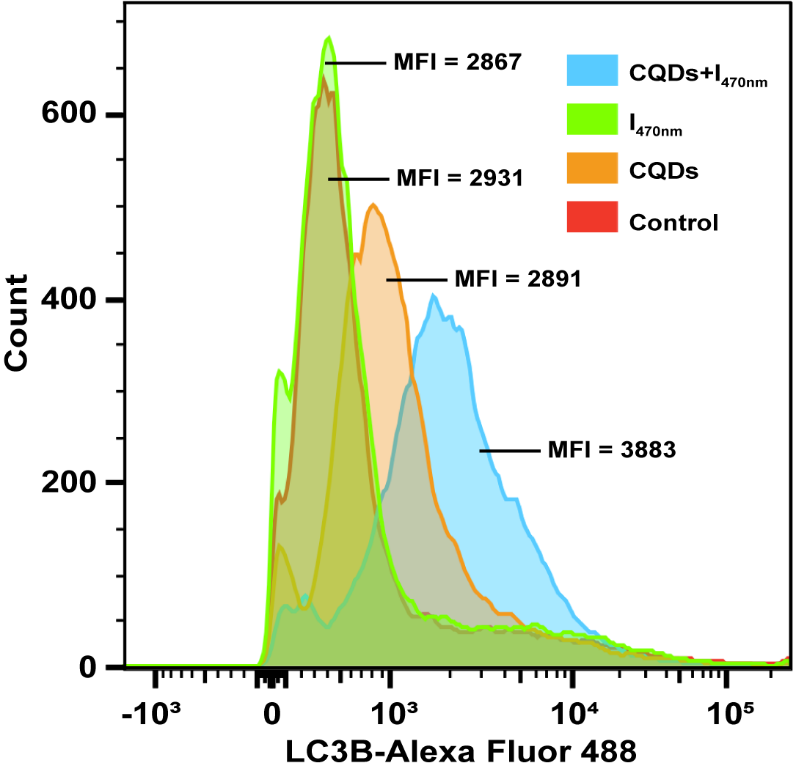


Figure S8. LC3B level detected by flow cytometry in Huh7 cells. Cells were treated with TC-WS-CQDs and/or exposed to 470 nm irradiation for 10 min. After 24 h, cells were labeled with anti-LC3B and Alexa Fluor 488 dye. MFI: Mean fluorescence intensity. CQDs+I_470nm_: TC-WS-CQDs with 470 nm irradiation, I_470nm_: 470 nm irradiation without TC-WS-CQDs, CQDs: TC-WS-CQDs, Control: no TC-WS-CQDs and irradiation.


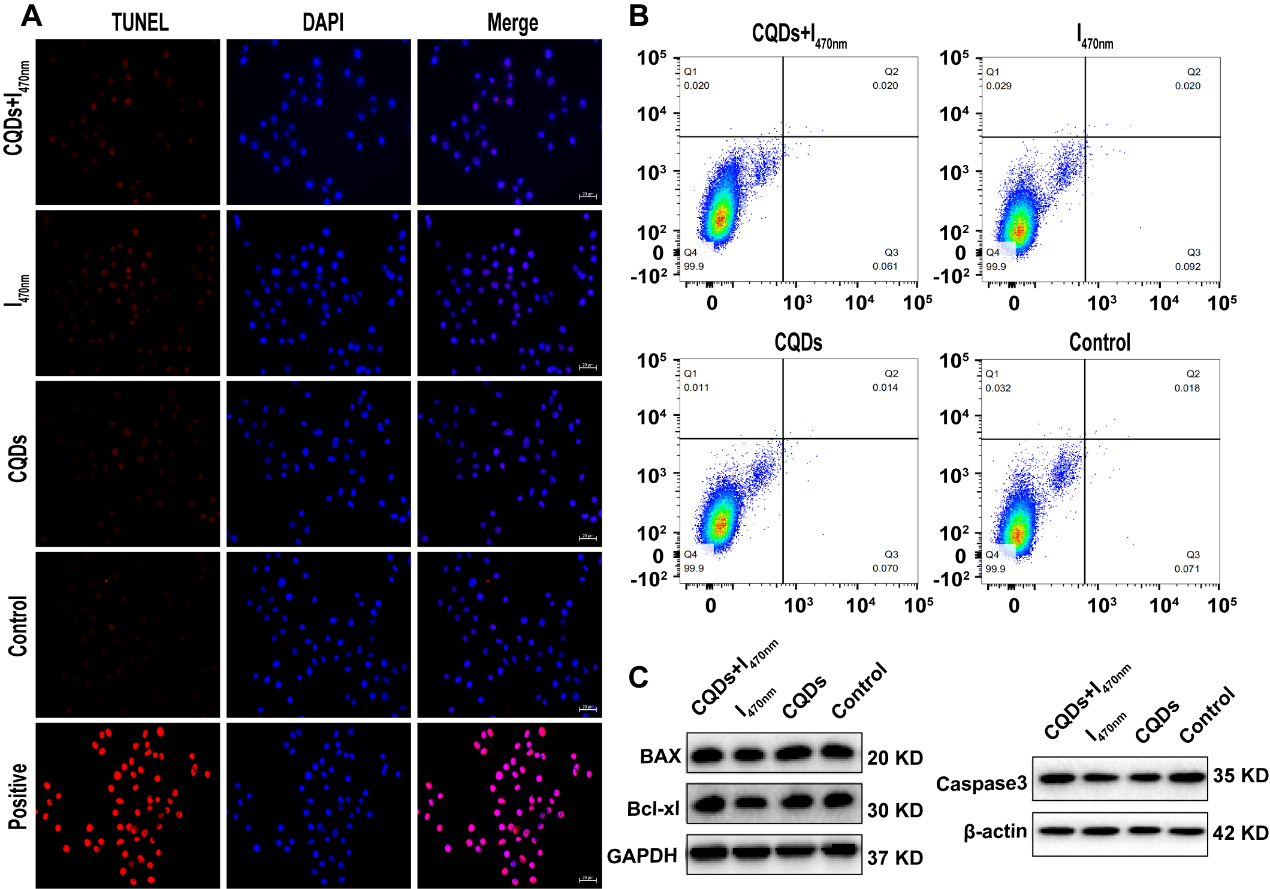


Figure S9. The test of apoptosis by 470 nm photoexcited TC-WS-CQDs in Huh7 cells. (A) TUNEL staining after different treatment. DNase I as the positive group. (B) FITC-PI/Annexin V staining determination apoptosis in Huh7 cells. (C) Western blot analysis the protein expressions of BAX, Bcl-xl, and Caspase3 after different treatment. CQDs+I_470nm_: TC-WS-CQDs with 470 nm irradiation, I_470nm_: 470 nm irradiation without TC-WS-CQDs, CQDs: TC-WS-CQDs, Control: no TC-WS-CQDs and irradiation.

**References:**

[1] H. Ding; S. Yu; J. Wei; H. Xiong. Full-Color Light-Emitting Carbon Dots with a Surface-State-Controlled Luminescence Mechanism. *Acs Nano* **2016**, 10, 484.

[2] J. Ge; Q. Jia; W. Liu; L. Guo; Q. Liu; M. Lan; H. Zhang; X. Meng; P. Wang. Red-Emissive Carbon Dots for Fluorescent, Photoacoustic, and Thermal Theranostics in Living Mice. *Adv. Mater.* **2015**, 27, 4169.

[3] C. L. Shen; Q. Lou; J. H. Zang; K. K. Liu; S. N. Qu; L. Dong; C. X. Shan. Near‐Infrared Chemiluminescent Carbon Nanodots and Their Application in Reactive Oxygen Species Bioimaging. *Adv Sci* **2020**, 7, 1903525.

[4] A. Nasrin; M. Hassan; V. G. Gomes. Two-photon active nucleus-targeting carbon dots: enhanced ROS generation and photodynamic therapy for oral cancer. *Nanoscale* **2020**, 12, 20598.

[5] M. Zheng; S. Ruan; S. Liu; T. Sun; D. Qu; H. Zhao; Z. Xie; H. Gao; X. Jing; Z. Sun. Self-Targeting Fluorescent Carbon Dots for Diagnosis of Brain Cancer Cells. *Acs Nano* **2015**, 9, 11455.
